# Improved Procedure for the 3D Reconstruction of Asphalt Concrete Mesostructures Considering the Similarity of Aggregate Phase Geometry between Adjacent CT Slices

**DOI:** 10.3390/ma16010234

**Published:** 2022-12-27

**Authors:** Chao Wang, Hui Xu, Yan Zhang, Yiren Sun, Weiying Wang, Jingyun Chen

**Affiliations:** 1 School of Transportation and Logistics, Dalian University of Technology, Dalian 116024, China; 2 City Institute, Dalian University of Technology, Dalian 116600, China; 3 College of Transportation Engineering, Tongji University, Shanghai 201804, China

**Keywords:** asphalt concrete, 3D reconstruction, digital image processing (DIP), computed tomography (CT), mesostructure

## Abstract

Existing image segmentation algorithms used for the computed tomography (CT) images of asphalt concrete mostly ignore the similarity of aggregate phase geometry between adjacent CT slices, thus increasing the variability in the aggregate phase pixel values between adjacent slices and leading to a large number of model defects, e.g., interconnected aggregates, flaky aggregates, and incomplete aggregates. The developed mesostructural models with these defects pose a challenge to following simulation operations. To address this issue, an improved procedure for the 3D reconstruction of asphalt concrete mesostructures considering the similarity of aggregate phase geometry between adjacent slices was developed, which includes two adjacent-slice pixel-value-correction algorithms, a multi-directional multiple-correction method, and an image pixel interpolation process. First, the bilinear interpolation algorithm was employed to improve the pixel density of 2D CT images and the average filtering algorithm was used to reduce the noise of the CT images. Subsequently, the OTSU method was employed to separate the asphalt mortar matrix phase from the aggregate phase, and the marker-based watershed segmentation method was used to separate the interconnected aggregates. Finally, the adjacent-slice pixel-value-correction algorithm was used to recover the similarity of aggregate phase geometry between adjacent CT slices, and the multi-directional multiple-correction method was used to further enhance the geometric similarity. The results show that the developed 3D reconstruction procedure removes most of the model defects in the 3D mesostructural model of asphalt concrete, thus realistically maintaining the 3D spatial distribution features and contour characteristics.

## 1. Introduction

In traditional pavement analysis and design, asphalt concrete is often considered a homogeneous material and its mechanical properties can be determined using relevant laboratory tests [1,2]. However, asphalt concrete is essentially a heterogeneous material, which actually contains the aggregate phase, asphalt mortar matrix phase, and air void phase at the mesoscale [3,4], and thus its overall mechanical behavior is closely associated with the interaction between various components in the mesostructure.

Numerical simulation approaches, such as discrete element (DE) and finite element (FE) methods, have been applied to investigate the mesomechanical behavior of asphalt concrete; however, a reliable mesostructural model of asphalt concrete needs to be realistically established before using these numerical methods to deeply explain the mechanical behavior and damage evolution mechanism of asphalt concrete at the mesoscale. Currently, the methods for establishing asphalt concrete mesostructures are classified into two major categories, namely the random aggregate generation method [5,6,7,8] and the CT-based digital image processing (DIP) technique [9,10,11]. Compared with the random aggregate generation method, the CT-based DIP technology can better reflect the real mesostructural morphological characteristics and spatial distribution of aggregates. Two types of CT-based DIP approaches, i.e., the 2D and 3D methods, are involved. The 2D methods are now more widely used in the asphalt paving community due to their simplicity in simulation. However, the 2D methods cannot reasonably explain the mechanical behavior because aggregates in asphalt concrete actually interlock with each other in a 3D fashion. In the common procedure for 3D CT image processing, filtering algorithms are first employed to reduce the noise of the 2D CT slice grayscale images. Then, image segmentation algorithms are adopted to segment the aggregate phase and the asphalt mortar matrix phase in 2D grayscale images to obtain 2D binary images. Finally, the processed 2D binary images of asphalt concrete are reconstructed into a 3D mesostructural voxel model along the CT acquisition direction. In this procedure, image segmentation is a key step that aims to facilitate the development of a geometrically reasonable 3D model by separating the aggregate phase from the asphalt mortar phase in 2D slices and eliminating interconnected aggregates in 2D slices. In the subsequent simulation, using these geometrically reasonable 3D numerical models will help improve the modeling accuracy and ensure computational convergence.

Among image segmentation techniques, the manual threshold selection (TH) [12] is the most basic one. The efficacy of this method is limited by the skill proficiency of the operator and it cannot guarantee the reproducibility of the image processing results. Therefore, it is not suitable for the high-volume image processing task required for 3D reconstruction [13]. OTSU is an automatic threshold determination method [14]. This method first assumes a threshold value in the grayscale range of image pixels, then classifies the pixels in the grayscale image into target and background categories according to the assumed threshold value, and finally uses the variance of the grayscale values of the two categories as the judgment index to obtain the best threshold value for this image by exhaustively searching for the maximum value of the variance [15,16]. Gong et al. [17] developed a 2D OTSU method. This method improves the segmentation quality of low signal-to-noise ratio images by simultaneously considering the gray values of image pixels and their 2D coordinate information [18]. Bhandari et al [19] developed the 3D-OTSU method. This method simultaneously considers the pixel gray value, neighborhood pixel gray median, and neighborhood pixel gray average to improve the segmentation quality of the image with a low signal-to-noise ratio and low contrast [20].

Although both the TH and OTSU methods can segment the aggregate phase and asphalt mortar matrix phase in the 2D CT slice images of asphalt concrete, a large number of model defects can be found in the obtained 3D model, such as interconnected aggregates, flaky aggregates, and incomplete aggregates, after 3D reconstruction is performed on the CT slice sets that have undergone the 2D image segmentation. Actually, there are huge differences in aggregate morphology for 2D slices and 3D models. The main reason for this discrepancy is that existing studies mostly ignore the connection of the pixels characterizing the aggregate phase between the adjacent slices in the procedure of obtaining optimal 2D binary image segmentation results, which inevitably increases the geometric differences in the aggregate phases between adjacent slices, and the accumulation of such geometric differences eventually leads to a large number of model defects in the constructed 3D voxel models. These model defects can adversely impact the convergence of simulation operations and even the correctness of simulation results. Due to the presence of numerous aggregates in the asphalt concrete mesostructural model, these defects are difficult to remove manually using software.

In the set of asphalt concrete CT slices, the 2D geometry and location information of the same aggregate possesses a natural similarity between adjacent slices. From a pixel perspective, this connection manifests itself as a continuity of pixel information at the same coordinates between adjacent slices. In order to overcome the above-mentioned model defects, this study developed a new procedure for the 3D reconstruction of asphalt concrete mesostructures considering the similarity of aggregate phase geometry between adjacent slices, which includes two adjacent-slice pixel-value-correction algorithms, a multi-directional multiple-correction method, and an image pixel density increase process.

## 2. Methodology

### 2.1. Preparation of Asphalt Concrete Specimens

In this study, asphalt concrete was considered as a two-phase particulate composite consisting of coarse aggregates (greater than 2.36 mm) and an asphalt mortar matrix containing the remaining fine aggregates, mineral fillers, and air voids.

To demonstrate the effectiveness of the developed procedure for the 3D reconstruction of asphalt concrete mesostructures, asphalt concrete specimens with a nominal maximum aggregate size (NMAS) of 13.2 mm were prepared. The asphalt used was PG 58–22 binder. The asphalt mix with an asphalt content of 3.88% was compacted using a Superpave gyratory compactor (Pine, Grove City, PA, USA). Finally, the compacted asphalt concrete specimens were cut into cylindrical specimens with a diameter of 65 mm and a height of 75 mm with an air void content of 4 ± 1%. Detailed information on the aggregate gradations of the asphalt concrete and asphalt mortar can be found elsewhere [21].

### 2.2. CT Image Acquisition

To obtain the mesostructural information of asphalt concrete, CT scanning was performed on cylindrical asphalt concrete specimens. The CT scanning equipment employed was the Germany Diondo d2 universal (Diondo, Hattingen, Germany)micro nano focus CT system with a 270 kV and 72 µA X-ray source. A set of 2D slice images (stack) representing the asphalt concrete mesostructure was reconstructed using the software XMReconstructor coupled to the micro-CT system. The stack displayed different grayscales in its 2D slice images. The slice spacing was 0.1 mm, with a resolution of 78 µm/pixel, and a total of 750 slices were displayed at 3.94 MB per slice.

The complete sample information of the asphalt concrete specimen consisted of 750 8-bit slices of 1507 pixels × 914 pixels. In each CT slice, pixels of different grayscale intensity levels between 0 (black) and 255 (white) represent different components. Higher-density components tend to yield higher grayscale intensities. In this way, the distribution of materials in the sample can be well identified based on the grayscale intensity values.

### 2.3. Bilinear Interpolation Algorithm

**Figure 1:**
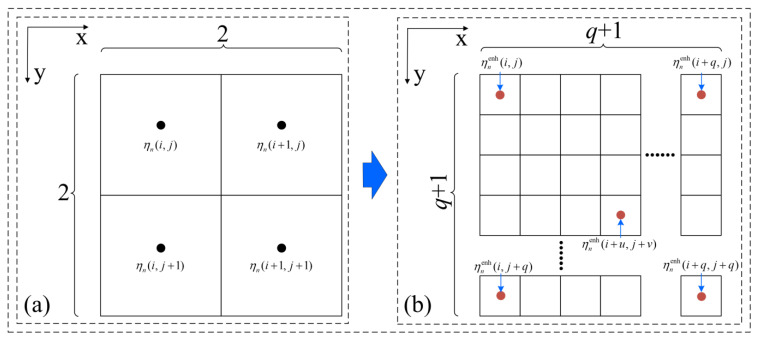
Schematic diagram of the bilinear interpolation algorithm: (**a**) before interpolation; (**b**) after interpolation.

To avoid the jagged distortion of the aggregate contours in CT slices during 2D image segmentation, the bilinear interpolation algorithm was used, which can increase the pixel density of an image without changing the image size. The interpolation process is shown in Figure 1.

Assuming that the pixel number of the image after pixel filling is *k*^2^ times the pixel number of the image before pixel filling:

(1) $$k=\frac{q+1}{2}$$

(2) $$\eta_{n}^{\mathrm{enh}}(i+u,j+v)=(1-\frac{u}{q})(1-\frac{v}{q})\eta_{n}(i,j)+(1-\frac{u}{q})\frac{v}{q}\eta_{n}(i,j+1)+\frac{u}{q}(1-\frac{v}{q})\eta_{n}(i+1,j)+\frac{u}{q}\frac{v}{q}\eta_{n}(i+1,j+1)$$

where $\eta_{n}^{\cdot }$ is the grayscale value of the pixel point in the *n*th slice before pixel interpolation; $\eta_{n}^{\mathrm{enh}}$ is the grayscale value of the new pixel point in the *n*th slice after pixel interpolation; *q* + 1 is the number of pixels in horizontal or vertical orientation after pixel interpolation; *i* is the x-axis pixel coordinate of the corresponding known pixel point; *j* is the y-axis pixel coordinate of the known pixel point; *u* is the x-axis coordinate of the new pixel point relative to the surrounding four known pixel points; and *v* is the y-axis coordinate of the new pixel point relative to the surrounding four known pixel points.

### 2.4. Two-Dimensional Image Segmentation Algorithm

In this study, the OTSU method was used to separate the aggregate phase from the asphalt mortar matrix phase, and then the marker-based watershed splitting method was used to split the interconnected aggregates.

**Figure 2:**
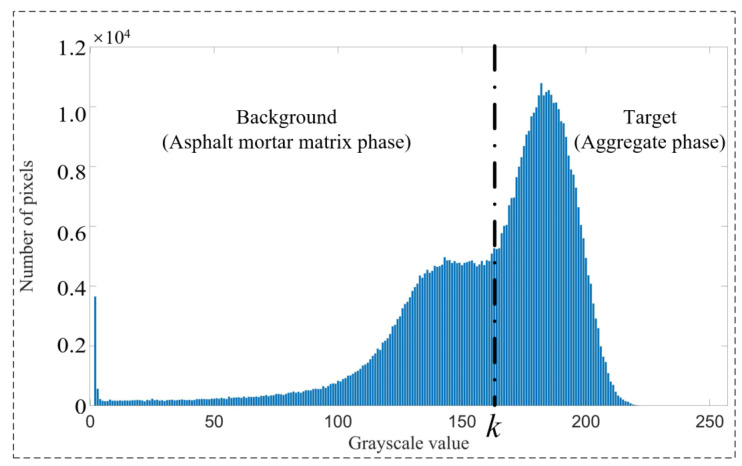
Schematic diagram of the OTSU method.

The OTSU method classifies the pixels in the grayscale image into two categories, target and background, according to the assumed threshold values, as shown in Figure 2. It first uses the variance of the grayscale values of the two categories as the judgment index to obtain the best threshold value for this figure by an exhaustive search and then transforms the grayscale image into a binary image containing the two categories of target (aggregate phase) and background (asphalt mortar matrix phase) according to the best threshold value.

The grayscale values of CT grayscale images range from 0 to 255, with a total of 256 grayscale levels. It is assumed that the number of pixels corresponding to the grayscale value *i* is $N_{i}^{\cdot }$; the optimal threshold is *k*; the grayscale value less than *k* is the background class; and the grayscale value greater than or equal to *k* is the target class. The interclass variance can be expressed as:

(3) $$\delta^{2}(k)=\omega_{\mathrm{back}}{\left[\mu -\mu_{\mathrm{back}}(k)\right]}^{2}+\omega_{\mathrm{targ}}{\left[\mu -\mu_{\mathrm{targ}}(k)\right]}^{2}$$

(4) $$\mu =\sum_{i=0}^{255}iP_{i}$$

(5) $$\mu_{\mathrm{back}}(k)=\frac{\sum_{i=0}^{k-1}iP_{i}}{\sum_{i=0}^{k-1}P_{i}}$$

(6) $$\mu_{\mathrm{targ}}(k)=\frac{\sum_{i=k}^{255}iP_{i}}{\sum_{i=k}^{255}P_{i}}$$

(7) $$\omega_{\mathrm{back}}(k)=\sum_{i=0}^{k-1}P_{i}$$

(8) $$\omega_{t\arg}(k)=\sum_{i=k}^{255}P_{i}$$

(9) $$P_{i}=\frac{N_{i}}{\sum_{i=0}^{255}N_{i}}$$

where *δ*^2^(*k*) is the corresponding interclass variance when the optimal threshold is assumed to be *k*; *μ* is the mean gray value of the whole CT slice; *μ*_back_(*k*) is the mean gray value of the corresponding background class pixels when the optimal threshold is assumed to be *k*; *μ*_targ_(*k*) is the mean gray value of the corresponding target class pixels when the optimal threshold is assumed to be *k*; *ω*_back_(*k*) is the ratio of the total number of background class pixels to the total number of CT slice pixels when the optimal threshold is assumed to be *k*; *ω*_targ_(*k*) is the ratio of the total number of target class pixels to the total number of CT slice pixels when the optimal threshold is assumed to be *k*; and *P_i_* is the ratio of pixels with gray value *i* to the total number of CT slice pixels.

At the maximum value max[*δ*^2^(*k*)], the optimal threshold value *k* is determined for this slice. At this point, the CT grayscale image will be transformed into a binary image containing only white (aggregate phase) and black (asphalt mortar phase):

(10) $$\varphi (x,y)=\left\{ \begin{array}{ll} 1 & i(x,y)\geq k\\ 0 & i(x,y)<k \end{array}\right.$$

where *φ*(*x,y*) is the binary value of the corresponding coordinate of the binary image after segmentation; *i*(*x,y*) is the gray value of the corresponding coordinate of the grayscale image before segmentation; *x* is the horizontal coordinate corresponding to the pixel in the CT slice image; and *y* is the vertical coordinate corresponding to the pixel in the CT slice image.

The marker-based watershed segmentation method was applied to segment the interconnected aggregates. By means of Matlab programming, the binary image was first subjected to limit erosion operations to obtain the internal markers of the aggregates. Then, the middle point of the aggregate-to-aggregate contact was calculated as the external markers using the distance function, and the 0-value pixels were used to connect the external markers at different locations as watershed ridges to obtain the watershed ridge segmentation layer. Finally, the watershed ridge segmentation layer was superimposed on the corresponding binary image to separate the inner markers and complete the segmentation of interconnected aggregates.

### 2.5. Developed Adjacent-Slice Pixel-Value-Correction Algorithms

From the pixel perspective, the pixel value distribution of the same aggregate in the CT slice set of asphalt concrete specimens has a natural similarity between adjacent CT slices due to the continuity of geometry. Based on this continuity of pixel values in adjacent layers in the CT slice set, two adjacent-slice pixel-value-correction algorithms are proposed in this paper.

Refer to the direction perpendicular to the CT slice plane as the CT acquisition direction, and define this direction as the Z-axis. Take the set of CT binary images that have experienced 2D image segmentation as the initial CT slice set, whose pixel information in each CT slice can be expressed as:

(11) $$\varphi_{n}(x,y)=\left\{ \begin{array}{l} 0\\ 1 \end{array}\right.$$

where *n* is the serial number of the CT slice in the acquisition direction (Z-axis) (the value range is 1–750); *x* is the coordinate of the horizontal corresponding to the pixel in the CT slice; *y* is the coordinate of the vertical corresponding to the pixel in the CT slice.

Depending on the processing purpose, this paper provides two adjacent-slice pixel-correction algorithms. It should be noted that all slices in the initial slice set need to have a uniform image size and pixel density.

Algorithm 1: First, take the intersection of adjacent-slice aggregate phase pixels in the initial slice set. Then, process the intersection of adjacent-slice aggregate phase pixels by morphological expansion operations. Finally, record the processed results in a new slice.

Algorithm 2: Firstly, take the union of adjacent-slice aggregate phase pixels of the initial slice set. Then, process the union of adjacent-slice aggregate phase pixels by morphological erosion operations. Finally, record the processed results in a new slice.

Apply Algorithm 1 or Algorithm 2 to all adjacent slices in the initial slice set to obtain a new slice set with one round of correction completed. Algorithm 1 is suitable for removing model defects in the 3D mesostructural model of asphalt concrete that cannot be well handled in the 2D image segmentation process, e.g., interconnected aggregates, flaky aggregates, and incomplete aggregates. Algorithm 2 is suitable for repairing the loss of pixel information in the outer contours of aggregates during processing. A detailed description of the two adjacent slice pixel correction algorithms is given as follows.

#### 2.5.1. Algorithm 1: First Take the Intersection and Then Perform Morphological Expansion (IMEX)

The intersection of the nth slice *φ_n_* and (*n* + 1)th slice *φ_n_*_+1_ in the initial slice set is taken, and the result of the operation is recorded in a new slice $\varphi_{n}^{I}$. The pixel values of this slice can be expressed as:

(12) $$\varphi_{n}^{I}(x,y)=[\varphi_{n}(x,y)]\cap [\varphi_{n+1}(x,y)]$$

**Figure 3:**
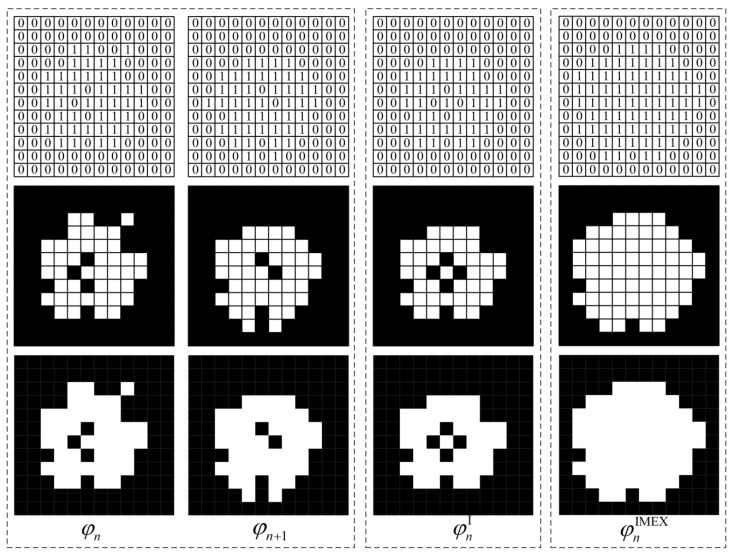
Schematic diagram of Algorithm 1 (IMEX).

Figure 3 illustrates the computational process of the IMEX algorithm from the pixel level. It can be found that after performing the intersection-taking operation, the overlapping region of the aggregate phases in the adjacent slices is recorded into the new slice $\varphi_{n}^{I}$, while the non-overlapping region is deleted. Therefore, the new slice $\varphi_{n}^{I}$ will lose a small amount of pixel information in the aggregate phase. In order to ensure the original volume fraction of aggregates in asphalt concrete without significant changes, it is necessary to take morphological expansion operations for the aggregate phase of the new slice, and the processed result is denoted as $\varphi_{n}^{\mathrm{IMEX}}$. The 2D four-connected domain kernel function (0 1 0, 1 1 1, 0 1 0) was chosen as the morphological operator in this study.

#### 2.5.2. Algorithm 2: First Take the Union and Then Perform Morphological Erosion (UMER)

The union of the *n*th slice *φ_n_* and (*n* + 1)th slice *φ_n_*_+1_ in the initial slice set is taken, and the result of the operation is recorded in a new slice $\varphi_{n}^{U}$. The pixel values of this slice can be expressed as:

(13) $$\varphi_{n}^{U}(x,y)=[\varphi_{n}(x,y)]\cup [\varphi_{n+1}(x,y)]$$

**Figure 4:**
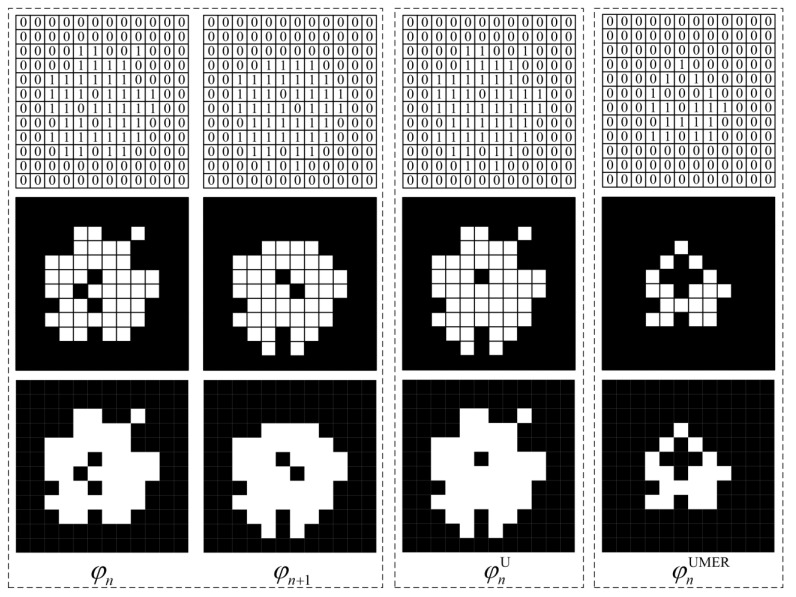
Schematic diagram of Algorithm 2 (UMER).

Figure 4 illustrates the computational process of the UMER algorithm from the pixel level. It can be found that after performing the union-taking operation, the overlapping region and non-overlapping region of the aggregate phases in the adjacent slices are recorded into the new slice $\varphi_{n}^{U}$. Therefore, the new slice $\varphi_{n}^{U}$ will add a small amount of pixel information from the aggregate phase. To ensure the original volume fraction of the aggregates in the asphalt concrete and reduce the interconnected aggregates, it is necessary to take morphological erosion operations for the aggregate phase of the new slice. The processed result is denoted as $\varphi_{n}^{\mathrm{UMER}}$.

#### 2.5.3. Multiple Correction

**Figure 5:**
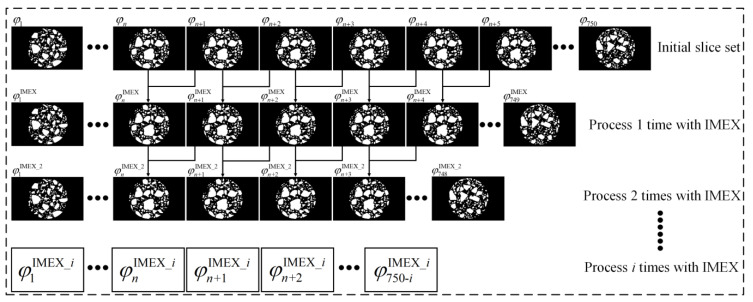
Schematic diagram of the multiple-correction method.

As described above, two adjacent-slice pixel-value-correction algorithms are proposed. After one round of correction processing, the aggregate phase in the new slice ($\varphi_{n}^{\mathrm{IMEX}}$ or $\varphi_{n}^{\mathrm{UMER}}$) integrates the aggregate phase morphological features of the *n*th slice *φ_n_* and the (*n* + 1)th slice *φ_n_*_+1_ in the initial slice set. Taking the IMEX algorithm as an example, the multiple-correction process is shown in Figure 5. After two rounds of correction processing, the aggregate phase in the new slice $\varphi_{n}^{\mathrm{IMEX}\_2}$ integrates the aggregate phase morphological features of *φ_n_*, *φ_n_*_+1_, and *φ_n_*_+2_ in the initial slice set. Therefore, it can be expected that as the number of multiple corrections (*i*) increases, the new slice $\varphi_{n}^{\mathrm{IMEX}\_i}$ obtained will integrate more information from the initial slice set and the recovery effect of the similarity of aggregate phase geometry between adjacent CT slices will be enhanced. However, 1-value pixels that distribute interruptedly along the acquisition direction in the slice set can become linear aggregates due to overcorrection. A limit should be set on the number of multiple corrections to avoid the appearance of linear aggregates. This limit is related to the slice spacing of the slice set, and the limit of multiple corrections is 10 in this paper.

It should be noted that after each round of correction processing, the pixel value of the slice at the end of the previous slice set is lost. The slice spacing chosen in this paper is 0.1 mm, i.e., the slice lost in each round of the correction process corresponds to the voxel value information of 0.1 mm thickness at the edge of the model. This model voxel loss is extremely small and thus negligible under the premise of limiting the number of multiple corrections.

#### 2.5.4. Multi-Directional Multiple Correction

Based on the Matlab toolbox, the binary slice set of the asphalt concrete can be obtained from any other direction by using the numerical model re-slicing algorithm and the 3D voxel reconstruction algorithm to simulate the CT nondestructive scanning process.

**Figure 6:**
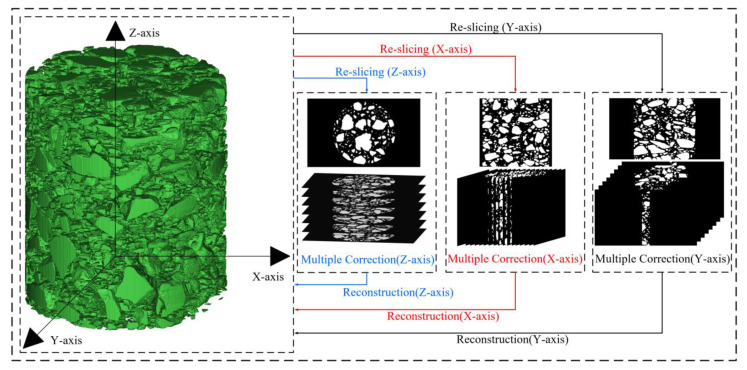
Schematic diagram of the multi-directional multiple-correction method.

As shown in Figure 6, multi-directional multiple correction is a combination of single-directional multiple-correction processes in multiple new directions. The single-directional multiple-correction process is to first re-slice the asphalt concrete 3D voxel model along a new direction to obtain a new slice set. Then, a multiple-correction process, as described above, is applied to the new slice set to recover and strengthen the similarity of aggregate phase geometry between the adjacent new slices. Finally, to facilitate the implementation of the multiple corrections in the next new direction, the corrected new slice set is reconstructed in 3D to obtain the new asphalt concrete mesostructural model.

The numerical model re-slicing algorithm converts the voxel values *ψ*(*x*,*y*,*z*) with 3D coordinate information to the pixel values *φ_x_*(*y*,*z*), *φ_y_*(*x*,*z*) or *φ_z_*(*x*,*y*) with 2D coordinate information along a certain direction and then arranges the pixel values by coordinates to generate a 2D slice set. Conversely, the 3D voxel reconstruction algorithm converts the pixel values *φ_x_*(*y*,*z*), *φ_y_*(*x*,*z*), or *φ_z_*(*x*,*y*) along the acquisition direction to obtain the voxel values, *ψ*(*x*,*y*,*z*), and then arranges the voxel values by coordinates to generate a 3D model. The parameter relationships are as follows:

(14) $$\psi (x,y,z)=\left\{ \begin{array}{l} \varphi_{x}(y,z)\\ \varphi_{y}(x,z)\\ \varphi_{z}(x,y) \end{array}\right.$$

where *ψ* is a voxel value with 3D coordinate information; *φ_x_* is a pixel value with 2D coordinate information in a slice perpendicular to the x-axis; *φ_y_* is a pixel value with 2D coordinate information in a slice perpendicular to the y-axis; and *φ_z_* is a pixel value with 2D coordinate information in a slice perpendicular to the z-axis.

### 2.6. Developed Procedure for the 3D Reconstruction of Asphalt Concrete Mesostructure

The bilinear interpolation algorithm introduced in Section 2.3 was employed to improve the pixel density of 2D CT images and the average filtering algorithm was used to reduce the noise of CT images. Subsequently, the OTSU method described in Section 2.4 was employed to separate the asphalt mortar matrix phase from the aggregate phase, and the watershed segmentation method based on markers was used to separate the interconnected aggregates. Finally, the IMEX and the UMER proposed in Section 2.5.1 and Section 2.5.2 was employed to recover the similarity of aggregate phase geometry between adjacent slices, and the recovery effect of geometric similarity was enhanced through the multi-directional multiple-correction method developed in Section 2.5.4.

**Figure 7:**
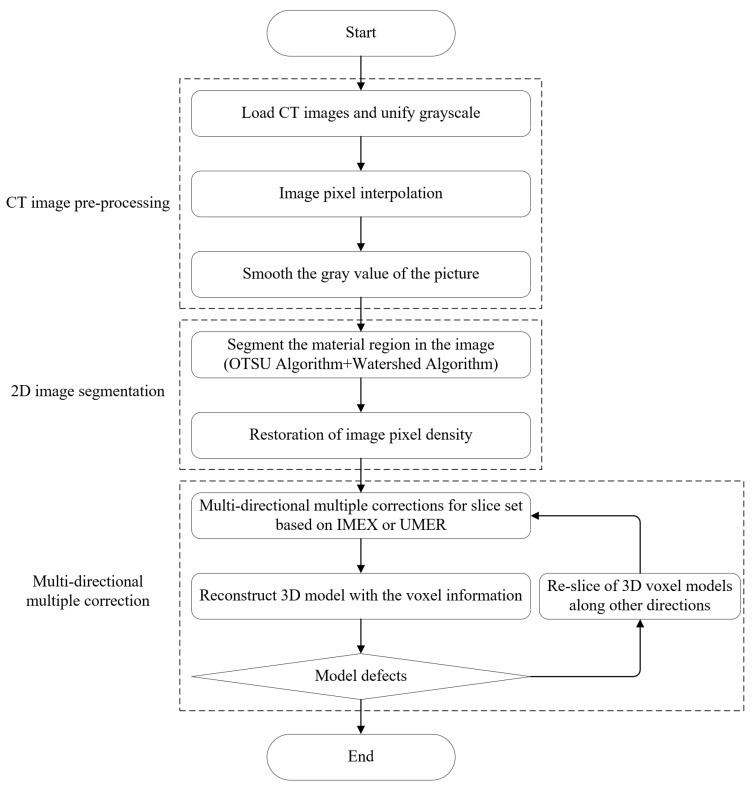
Flow chart of the improved procedure for the 3D reconstruction of an asphalt concrete mesostructure considering the similarity of aggregate phase geometry between adjacent slices.

Figure 7 shows the details of the improved procedure for 3D reconstruction. It includes CT image preprocessing (image pixel interpolation and image filtering), 2D image segmentation (OTSU image segmentation, watershed segmentation, and image pixel density restoration), and multi-directional multiple correction (re-slicing, multiple correction, and voxel reconstruction).

The workstation configuration for performing the 3D reconstruction process included an Intel® Core TM i7-9700 3.0GHz processor (Dell, Lundrock, TX, USA), 64GB RAM (DDR4), and operating system version Windows 10 19044.2006 (Professional Edition), and the processing software version is Matlab 2021.

**Figure 8:**
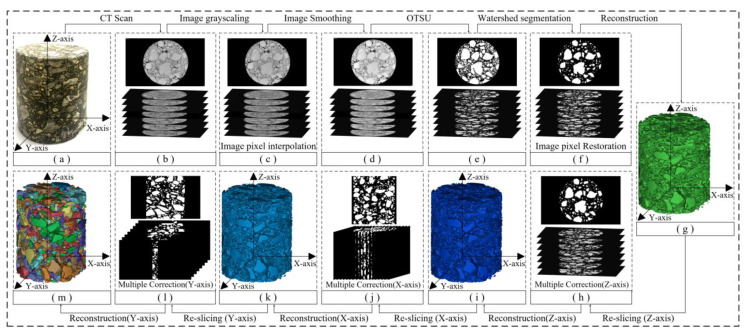
Processing results of each stage of the improved procedure for 3D reconstruction: (**a**) asphalt concrete specimen; (**b**) original CT images of asphalt concrete specimen; (**c**) CT images after grayscaling and pixel interpolation; (**d**) CT images after smoothing; (**e**) CT images after the OTSU segmentation; (**f**) CT images after the watershed segmentation and pixel restoration; (**g**) Completing the 3D voxel reconstruction; (**h**) Completing the Z-axis multiple correction; (**i**) Completing the 3D voxel reconstruction along the Z-axis; (**j**) Completing the X-axis multiple correction; (**k**) Completing the 3D voxel reconstruction along the X-axis; (**l**) Completing the Y-axis multiple correction; (**m**) Completing the 3D voxel reconstruction along the Y-axis.

According to the new process of 3D reconstruction given in Section 2.6, this study provides the processing results of each stage of 3D reconstruction, as shown in Figure 8.

The CT image pre-processing process is shown in Figure 8b–d. First, the CT slice set image information was read and the CT slice set was uniformly grayed out to obtain the CT grayscale slice set in order to avoid image format transformation due to file dumping.

Then, the pixel density of the CT grayscale slice set was increased based on the bilinear interpolation algorithm to prevent the contour distortion of the aggregate phase caused by image filtering and 2D image segmentation.

**Figure 9:**
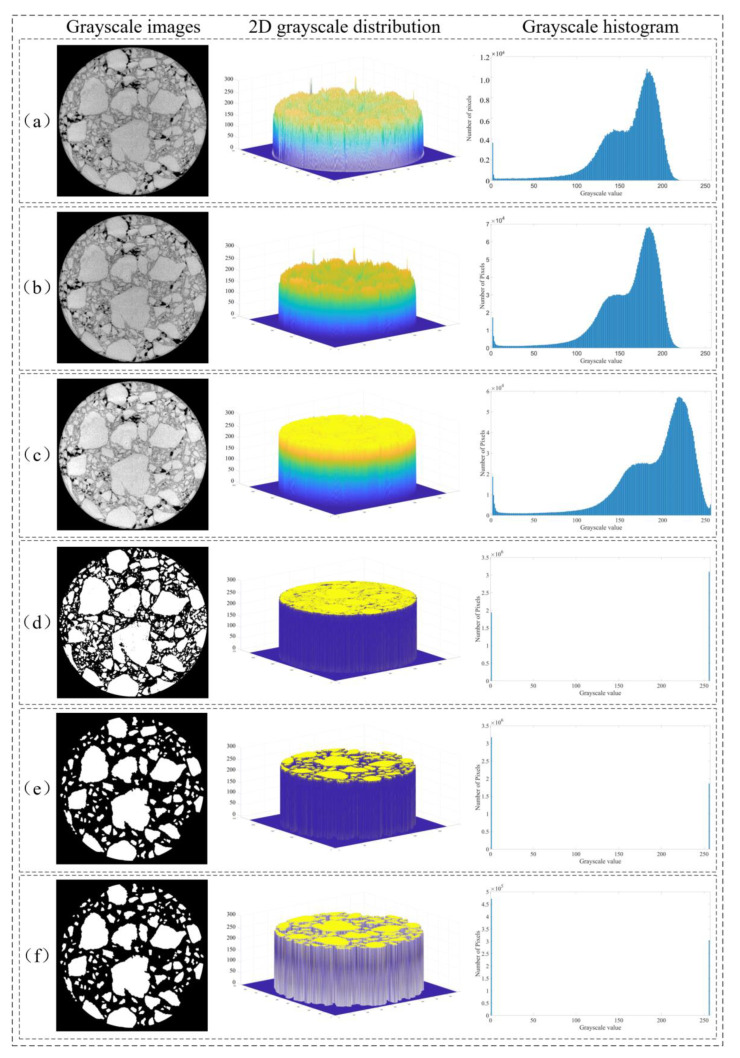
Changes in the grayscale of CT slice images during CT image preprocessing and 2D image segmentation stages: (**a**) after uniform grayscale; (**b**) after increasing image pixel density; (**c**) after the filtering process; (**d**) after OTSU segmentation; (**e**) after watershed segmentation; (**f**) after restoring image pixel density.

Finally, there was a loss of light intensity when the X-ray passes through the model because of the power limitation of CT. The overall grayscale of the CT grayscale image shows a distribution trend of low center and high surroundings (Figure 9a), which will reduce the ability of the numerical algorithm to recognize pixels representing different materials in CT grayscale slices. In this study, the average filtering algorithm was selected to filter the CT grayscale slices with high pixel density, which enhanced the global contrast of the pixel grayscale values between the asphalt mortar matrix phase and the aggregate phase in the CT grayscale slice (Figure 9c) and weakened the noise impact to improve the image segmentation quality of the OTSU algorithm for low-contrast images.

The 2D image segmentation process is shown in Figure 8d–f. First, the optimal threshold of each slice of the CT grayscale slice set was calculated based on the OTSU method’s exhaustive calculation, and the grayscale image was segmented into a binary image containing only the asphalt mortar matrix phase and the aggregate phase according to the optimal threshold (Figure 9d).

Then, the segmentation line layer was obtained by using the watershed segmentation method based on markers, and it was overlaid on the corresponding CT binary slice to complete the segmentation of the interconnected aggregates. At this time, a large number of randomly scattered 0-value or 1-value pixel blocks appeared in the CT binary slice, which mainly included fine aggregates with diameters less than 2.36 mm and erroneous data under the influence of noise could be removed using the image morphology method. The processing results are shown in Figure 9e.

Finally, to ensure that the voxel model obtained from the subsequent 3D reconstruction is consistent with the dimensions of the original specimen, the pixel density of the CT binary slice set was restored based on the inverse operation of the bilinear interpolation algorithm after the 2D image segmentation was completed (Figure 9f).

The multi-directional multiple-correction process of the asphalt concrete 3D voxel model is shown in Figure 8g–m. Based on the multi-directional multiple-correction method proposed in Section 2.5.4, the 3D voxel model of the asphalt concrete with model defects was corrected along the Z-axis, X-axis, and Y-axis successively.

## 3. Results and Discussion

**Figure 10:**
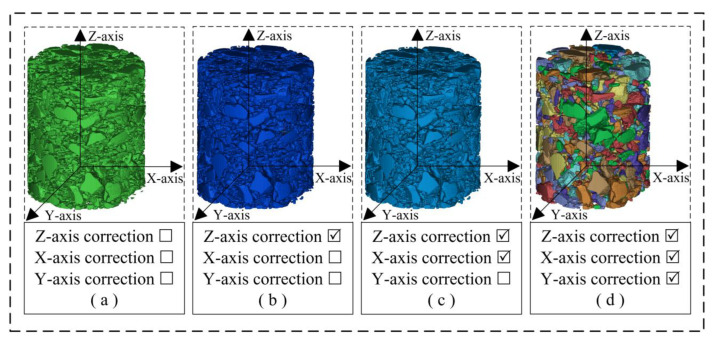
Three-dimensional voxel models at each stage of the improved procedure for 3D reconstruction: (**a**) Model A: obtained from 3D reconstruction of the CT slice set after 2D image segmentation; (**b**) Model B: Z-axis multiple correction completed on the basis of Model A; (**c**) Model C: X-axis multiple correction completed on the basis of Model B; (**d**) Model D: Y-axis multiple correction completed on the basis of Model C.

Figure 10 shows the 3D models at each stage under the improved procedure for 3D reconstruction. These models are discussed in detail in this section. Table 1 shows the main defects in the 3D model at each stage.

### 3.1. Two-Dimensional Slice Analysis

Using the numerical model re-slicing algorithm can obtain sections in any direction of the 3D model, which will facilitate the observation of the mesostructure of the asphalt mixture model.

**Figure 11:**
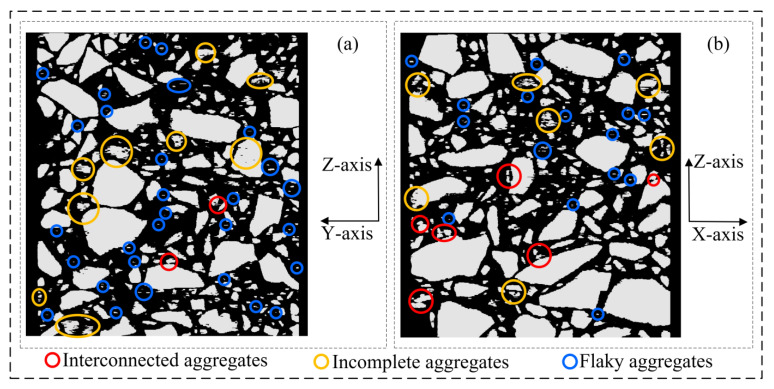
Asphalt concrete 3D voxel model sections (corresponding to Figure 10a): (**a**) YZ section; (**b**) XZ section.

As shown in Figure 11, the 3D voxel model (Figure 10a) was re-sliced to obtain its vertical sections (XZ section and YZ section). It can be found that the morphology of the aggregate phase in the vertical section differs greatly from that in the horizontal section (XY section). In the asphalt concrete voxel model obtained by the 3D reconstruction of the CT slice set that has completed 2D image segmentation, there actually exists a large number of flaky aggregates distributed perpendicular to the Z-axis (CT acquisition direction), and the problems of interconnected aggregates and incomplete aggregates are very serious.

**Figure 12:**
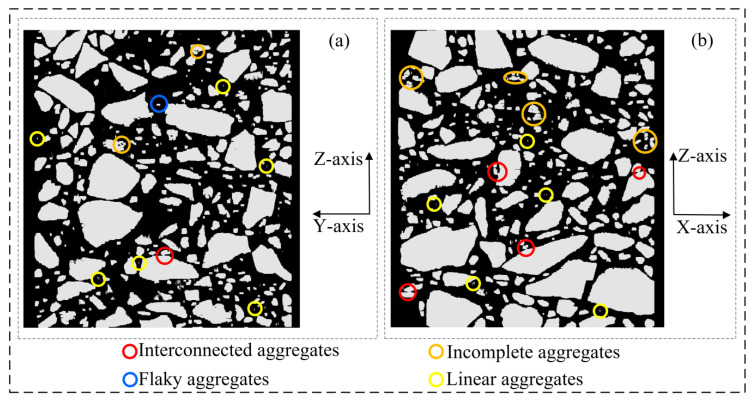
Three-dimensional asphalt concrete voxel model sections (corresponding to Figure 10b): (**a**) YZ section; (**b**) XZ section.

As shown in Figure 12, the 3D voxel model (Figure 10b) with the Z-axis multiple correction completed was re-sliced. In its vertical sections (XZ section and YZ section), it can be found that the Z-axis multiple correction is very effective in removing the flaky aggregates distributed perpendicular to the Z-axis, and the problems of incomplete aggregates and interconnected aggregates have been solved to some extent.

However, due to the cumulative effect of the multiple-correction algorithm in the correction direction, when the number of multiple corrections is high, 1-value pixels that distribute interruptedly along the acquisition direction in the slice set become linear aggregates distributed along the correction direction. It is verified that this linear aggregate will be automatically eliminated during subsequent corrections in other directions.

**Figure 13:**
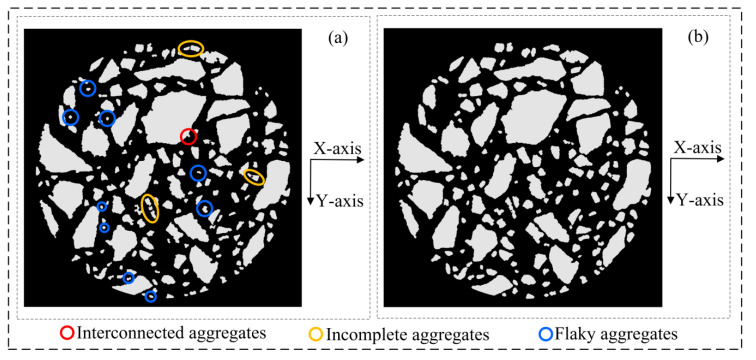
Asphalt concrete 3D voxel model sections (XY section): (**a**) before Z-axis multiple correction (corresponding to Figure 10a); (**b**) after Z-axis multiple correction (corresponding to Figure 10b).

As shown in Figure 13, the model before Z-axis multiple correction (Figure 10a) and the model after Z-axis multiple correction (Figure 10b) are re-sliced. In the XY sections of the two models, it can be found that although the sliced images processed by 2D image segmentation have good aggregate separation characteristics, there are actually a large number of model defects such as flaky aggregates, incomplete aggregates, and interconnected aggregates in the processed binary images due to the neglect of the similarity of aggregate phase geometry between adjacent slices. More importantly, the geometry and distribution of these model defects in 3D space are random in nature and cannot be directly confirmed from XY sections by visual comparison or 2D parametric characterization methods.

**Figure 14:**
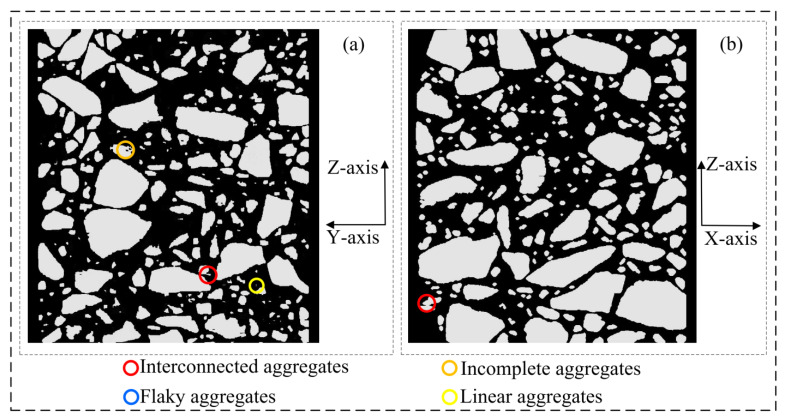
Asphalt concrete 3D voxel model sections (corresponding to Figure 10c): (**a**) YZ section; (**b**) XZ section.

As shown in Figure 14, in the XZ section and YZ section of the 3D voxel model (Figure 10c) that has completed X-axis multiple correction, it can be found that the linear aggregates parallel to the Z-axis distribution in the 3D voxel model (Figure 10b) have largely disappeared after the X-axis correction, but there is still a small number of interconnected aggregates and incomplete aggregates.

**Figure 15:**
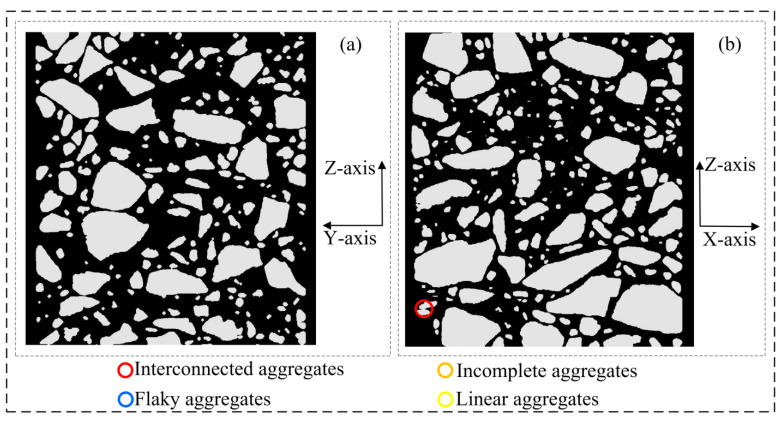
Asphalt concrete 3D voxel model sections (corresponding to Figure 10d): (**a**) YZ section; (**b**) XZ section.

As shown in Figure 15, in the XZ section and YZ section of the 3D voxel model (Figure 10d) that has completed the Y-axis multiple correction, it can be found that the model defects in the asphalt concrete voxel model have been well resolved at this time.

### 3.2. Three-Dimensional Model Analysis

**Figure 16:**
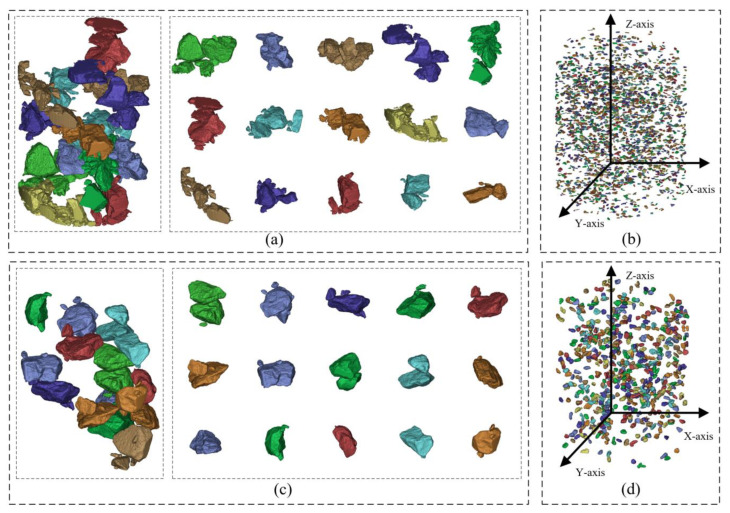
Aggregate extraction results for the 3D voxel model: (**a**) large volume aggregates without considering the similarity of aggregate phase geometry between adjacent slices (Figure 10a); (**b**) small volume aggregates without considering the similarity of aggregate phase geometry between adjacent slices (Figure 10a); (**c**) large volume aggregates considering the similarity of aggregate phase geometry between adjacent slices (Figure 10d); (**d**) small volume aggregates considering the similarity of aggregate phase geometry between adjacent slices (Figure 10d).

In the 3D voxel model, the aggregate volume can be determined based on the number of voxels contained in a single aggregate particle. The aggregates in the uncorrected 3D voxel model (Figure 10a) and the 3D voxel model that has completed multi-directional multiple correction (Figure 10d) are arranged in order of volume. The interconnected aggregates are multiple independent aggregates that are very close to each other and thus incorrectly identified as a single particle during image processing. They are generally larger in volume than real aggregates. To facilitate the presentation, large-volume aggregates (mainly interconnected aggregates) and small-volume aggregates (mainly flaky aggregates) are extracted from both the corrected and uncorrected voxel models. The extraction results are shown in Figure 16.

It can be found that in large-volume aggregates (Figure 16a,c), the 3D aggregates constructed by the procedure without considering the similarity of aggregate phase geometry between adjacent slices restore the 3D spatial distribution characteristics and contour characteristics of the real aggregates to a certain extent, but the model defect of interconnected aggregates is very serious and often appears along with flaky aggregates and incomplete aggregates, forming more complex comprehensive model defects. These comprehensive model defects will greatly increase the workload and difficulty of manual processing. In contrast, the aggregates constructed by the improved procedure for the 3D reconstruction of asphalt concrete mesostructures considering the similarity of aggregate phase geometry between adjacent slices have good 3D spatial distribution characteristics and contour characteristics, while the model defects are greatly reduced and the remaining small amount of interconnected aggregates can be easily solved by hand.

In small-volume aggregates (Figure 16b,d). A large number of independently distributed flaky aggregates exist in the fine aggregates constructed without considering the similarity of aggregate phase geometry between adjacent slices, and they are mostly distributed in the whole asphalt concrete model at an angle perpendicular to the Z-axis. In this paper, after the multiple correction of the 3D voxel model along Z-axis, the flaky aggregates are effectively eliminated and the real 3D spatial distribution information of a large number of small aggregates in the asphalt concrete is preserved, which is finally recovered to the small aggregates with real 3D contour characteristics after the X-axis multiple correction and Y-axis multiple correction.

**Figure 17:**
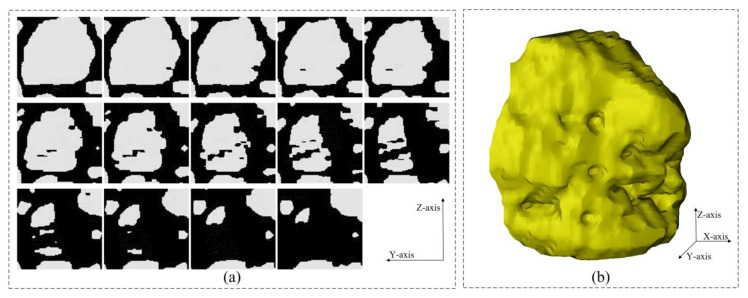
Schematic diagram of the concave contour of the aggregate: (**a**) 2D slice; (**b**) 3D model (Figure 10d).

Figure 17 shows the details of the concave contour of the 3D aggregate constructed by the improved procedure for 3D reconstruction and the 2D slice image corresponding to this contour. It can be found that the partially closed holes in the aggregate particles in the 2D slice result from slicing the 3D aggregate outer contour depression at a specific angle. Based on the proposed IMEX and UMER algorithms, these 2D closed holes were preserved by using the developed multi-directional multiple-correction method. The final 3D aggregate model obtained preserves the concave characteristics of the actual aggregate outer contour.

The geometric similarity between adjacent slices is naturally present and would not disappear due to the change in aggregate particle size or in material properties. Therefore, the developed procedure for 3D reconstruction is also effective for other asphalt concretes with different aggregate sizes, or other heterogeneous materials with significant density differences.

However, it should be noted that the greater the spacing between adjacent CT slices, the weaker the geometric similarity of adjacent CT slices. The developed procedure for 3D reconstruction requires that the spacing between adjacent CT slices should be less than or equal to 0.3 mm.

## 4. Summary and Conclusions

This paper develops an improved procedure for the 3D reconstruction of asphalt concrete mesostructures considering the similarity of aggregate phase geometry between adjacent slices, which includes two adjacent-slice pixel-value-correction algorithms, a multi-directional multiple-correction method, and an image pixel interpolation process. In the 3D reconstruction procedure, we used numerical algorithms to reproduce the natural geometric continuity of the asphalt concrete in order to eliminate model defects. The adjacent-slice pixel-value-correction algorithm was used to recover the similarity of aggregate phase geometry between adjacent CT slices, and the suggested multi-directional multiple-correction method was employed to further enhance the geometric similarity. The image pixel interpolation was applied to increase the image pixel density. Based on the analysis and results of this study, the following conclusions were drawn:

- Using the developed procedure for 3D reconstruction can efficiently eliminate the vast majority of model defects in the asphalt concrete mesostructural model.
- By means of the proposed adjacent slice pixel correction algorithms, the multiple corrections implemented along the CT acquisition direction can effectively remove the model defects (interconnected aggregates, incomplete aggregates, and flaky aggregates) distributed perpendicular to the CT acquisition direction in the 3D voxel model.
- Based on the proposed adjacent slice pixel correction algorithms, the closed holes in the 2D slice corresponding to the concave features of the 3D aggregate can be preserved by using the developed multi-directional multiple-correction method. The resulting 3D aggregate model will have the concave characteristics of the actual aggregate outer contour.
- The multi-directional multiple-correction method can more accurately evaluate the image segmentation effect of 2D slice images by acquiring 3D model slices in different directions.
- The image pixel interpolation process can increase the pixel density of the image to avoid the distortion of aggregate contours during 2D image segmentation.

Although this study demonstrates the effectiveness of the developed procedure for 3D reconstruction in removing model defects from the 3D mesostructure of asphalt concrete, it still cannot completely avoid manual processing; that is, the final modeling results are still influenced by subjective factors from the operator. In future studies, more effective algorithms should be designed to achieve the fully automated modeling of the 3D mesostructure of asphalt concrete, and the validity of the method will be verified using different asphalt concretes.

## Figures and Tables

**Table 1:** The main defects in the 3D model at each stage.

Model	Main Model Defects
A	Interconnected aggregates
	Incomplete aggregates
	Flaky aggregates
B	Interconnected aggregates
	Incomplete aggregates
	Linear aggregates
C	Interconnected aggregates
	Incomplete aggregates
D	Interconnected aggregates (few)

## Data Availability

Data sharing is not applicable to this article.
